# Ultrasonic treatment effects on hydroxyl radical generation in various solution systems: an iodometric analysis

**DOI:** 10.1016/j.ultsonch.2026.107917

**Published:** 2026-06-09

**Authors:** Yuanfang Liu, Yuanxiao Liu, Hailu Hou, Minghui Liu, Ying-Ying Li, Jinming Xu, Jiayu Guo

**Affiliations:** a Henan Engineering Technology Research Center for Green Catalytic and Atom Economic Conversion of Coal-based Benzene, Department of Chemistry, Zhengzhou Normal University, No. 6, Yingcai Street, Huiji District, Zhengzhou 450044, China; b College of Food Science and Technology, Henan Agricultural University, No. 63, Nongye Road, Jinshui District, Zhengzhou 450002, China; c Key Laboratory for Information System of Mountainous Areas and Protection of Ecological Environment, Guizhou Normal University, Guiyang 550001, China

**Keywords:** Apparent •OH yield, Ultrasonic treatment, Iodometry, Cavitation, Advanced oxidation technology, Sonochemical processes

## Abstract

Hydroxyl radicals (•OH) are key reactive species in sonochemical processes; however, their generation is strongly dependent on the operating parameters and solution composition. In this study, probe-ultrasonication-induced oxidizing response was evaluated using an iodometric method in a 0.4mol L^−1^ KI background, where the measured response reflects the accumulated titratable iodine (I_3_^-^) signal and is therefore discussed as an apparent •OH yield rather than an instantaneous steady-state [•OH]. The effects of sonication time (5–35 min), ultrasonic power (60–300 W), duty cycle (20%–100%), and probe position (top/middle/bottom) were systematically investigated. The apparent •OH yield increased with time and power, reached a maximum at a duty cycle of 60%, and was highest when the probe was positioned at the center of the liquid column. The influence of solution composition was further evaluated by adding ethanol, acetic acid, NaOH, NaHCO_3_, and NaCl to the KI background. Increasing the ethanol and NaOH concentrations generally decreased the apparent •OH yield, whereas acetic acid showed a biphasic response. NaHCO_3_ decreased the apparent •OH yield in a concentration-dependent manner, and NaCl showed a non-monotonic concentration dependence. In phosphotungstic acid systems, the iodometric response increased with concentration at 10 min but the concentration dependence diminished at 30 min, and a no-ultrasound control showed a measurable baseline signal while ultrasound produced a stronger response. Hydrogen peroxide markedly enhanced the response at moderate concentration but reduced the apparent •OH yield at higher levels due to competing reactions, while a t-BuOH scavenger control reduced the signal to about 10% of that without t-BuOH, indicating predominantly •OH-driven response with minor non-specific contributions. These results provide practical guidance for regulating the accumulated oxidative response in probe-ultrasonic systems.

## 1 Introduction

Ultrasonic treatment, a prominent advanced oxidation technology (AOT), has gained considerable attention owing to its extensive applications in diverse fields. In environmental engineering, ultrasound technology effectively facilitates wastewater degradation and gas purification [1]. Additionally, ultrasound is integral to diagnostic and therapeutic applications in industry and medicine, enhancing energy efficiency and promoting sustainable natural resource management, which is aligned with contemporary socio-economic developments [2]. Within the food industry, ultrasonic treatment significantly improves sensory characteristics, hardness, and texture, while simultaneously enhancing product quality and accelerating processing stages. Ultrasonic technology aids in preserving nutritional integrity and prolonging shelf life, offering a more practical alternative to traditional preservation methods that do not require additional chemical agents [3], [4]. Ultrasonic waves are generated by mechanical vibrations, causing the periodic displacement of particles within a medium. The resultant high energy ultrasound-medium interactions induce structural alterations. Specifically, ultrasonic wave propagation in aqueous media induces periodic cycles of compression and rarefaction, leading to cavitation, which is characterized by the formation and subsequent implosion of the bubbles. Cavitation conditions generate extremely localized temperatures of approximately 4000 K and pressures of up to 500 atm, catalyzing both physical and chemical transformations, known as cavitation effects [5]. These extreme conditions facilitate the dissociation of water molecules into hydrogen and hydroxyl radicals (·OH), thereby enabling the oxidation of recalcitrant organic compounds. The redox potential of • OH varies with pH: approximately 1.9–2.0 V at pH ≥ 7 and 2.4–2.8 V at pH < 7 [6]. • OH is a potent oxidant that aggressively acquires electrons from adjacent molecules. They are highly reactive and can participate in various transformation pathways; therefore, reliable evaluation and monitoring of the oxidative response under practical conditions is important. [7].

Owing to the highly reactive nature and short lifespan of ·OH, their concentrations can only be indirectly quantified by detecting specific adducts [8]. The methods employed for detection include spectrophotometry, fluorometry, electron spin trapping, high-performance liquid chromatography (HPLC), chemiluminescence, iodometry and electrochemical detection [7]. KI-based iodometry (Weissler dosimetry) has been widely used as a practical approach to evaluate accumulated oxidizing equivalents in sonochemical systems [9], [10], [11]. However, the scope and limitations of the Weissler reaction, especially in complex matrices where multiple oxidants and secondary radicals may contribute, have also been discussed [12]. Zhou Kaiwei developed a sensitive and stable electrochemical sensor utilizing CuO-CeO_2_/MXene to detect ·OH produced during the decomposition of hydrogen peroxide (H_2_O_2_). Although this sensor demonstrated remarkable sensitivity, stability, and reproducibility, its practical application is hindered by its limited resistance to interference, necessitating its integration with techniques such as chemiluminescence and capillary electrophoresis [13]. Jankov Milica employed high-efficiency thin-layer chromatography to assess the antioxidant capacity of Sempervivum tectorum L. leaf extracts, effectively comparing their radical scavenging abilities. Despite the notable efficiency and sensitivity of HPLC methods, their inherent complexity and susceptibility to interference from intermediates and by-products impede the accurate quantification of free radicals [14]. Fluorometric spectrophotometry, although cost-effective and operationally straightforward with adequate sensitivity for chemically induced fluorescence-targeted reactions, frequently encounters specificity limitations owing to interference from matrices and additives. Yang Qingxin [15] overcame this issue by exploiting a specific aminophenol-dopamine reaction to produce highly fluorescent nitrogen-heterocyclic compounds, enabling indirect detection of aminosalicylic acid. Additionally, Ri-Fu Yang [16] employed ultrasonic-assisted electrostatic field technology to evaluate the total flavonoid content in *Areca catechu*, validating the synergistic effects of ·OH and static fields using a simplified iodometric method. Given the simplicity and robustness of KI iodometry, we used it to evaluate the accumulated iodometric response, which is discussed as the apparent •OH yield rather than the instantaneous •OH concentration.

In the present investigation, an iodometric approach was utilized to explore the effects of variations in ultrasonic treatment parameters, including treatment time, power, duty cycle, and amplitude rod probe positioning on the apparent •OH yield. Specifically, the matrix-dependent trends of the apparent •OH yield in solutions containing ethanol, acetic acid, sodium hydroxide, sodium bicarbonate, sodium chloride, phosphotungstic acid, and H_2_O_2_ were systematically analyzed under controlled conditions. Although several trends align with established sonochemical behavior, this work provides a reproducible parametric map of iodometric response on a single probe-ultrasonic platform (fixed KI background, standardized titration, and matrix-matched blanks). The t-BuOH scavenger control and the no-ultrasound PTA control further define practical method boundaries and support interpreting the signal as an apparent •OH yield under the tested conditions.

## 2 Materials and methods

### 2.1 Materials

Analytical grade potassium iodide (KI) was purchased from Tianjin Jinbei Fine Chemical Co., Ltd. Sodium thiosulfate (Na_2_S_2_O_3_, analytical grade) was purchased from Beijing Beihua Fine Chemicals Co., Ltd. Soluble starch, anhydrous ethanol, sodium hydroxide, phosphotungstic acid, and hydrogen peroxide (30% w/w) were obtained from Tianjin Kaitong Chemical Reagent Co., Ltd. Sodium chloride was purchased from China Salt Industry Co., Ltd., and sodium bicarbonate was purchased from Tianjin Honglu Food Co., Ltd. Potassium iodate (KIO_3_, analytical grade) was used as the primary standard for standardizing the Na_2_S_2_O_3_ titrant. Distilled water was used for all the solution preparations. Ultrasonic irradiation was performed using a probe-type ultrasonic processor (JY92-IIN, Ningbo Scientz Biotechnology Co., Ltd., Ningbo, China).

### 2.2 Preparation of basic solutions

All solutions were prepared using distilled water at ambient temperature. Unless otherwise stated, concentrations expressed in % (w/v) refer to g per 100 mL solution, and % (v/v) refer to mL per 100 mL solution.

Potassium iodide (KI) solution (0.4mol L^−1^): KI (purity ≥ 99.0%) was dissolved in water and diluted to a volume to obtain a final concentration of 0.4mol L^−1^. The solution was then stored in an amber bottle. Sodium thiosulfate solution (Na_2_S_2_O_3_, 0.002mol L^−1^): Na_2_S_2_O_3_ was dissolved in water and diluted to volume to prepare a 0.002mol L^−1^ titrant solution. The titrant was standardized prior to use using potassium iodate (KIO_3_) as the primary standard (according to a standard iodometric procedure).

Starch indicator (1%, w/v): Soluble starch (1.0 g) was dispersed in a small volume of water to form a slurry, which was added to boiling water with stirring, boiled for 1–2 min, cooled, and diluted to 100 mL. The indicator was freshly prepared and stored at 4°C.

Preparation of test solution systems (in 0.4mol L^−1^ KI background):

To investigate the effects of different solution systems on apparent •OH yield during sonication, all test solutions were prepared in the KI background solution (0.4mol L^−1^) by adding the corresponding reagent and diluting to volume:

Ethanol solutions (% v/v): 0.5%, 1.0%, 2.0%, 3.0%, and 4.0% (v/v).

Acetic acid solutions (% v/v): 0.5%, 1.0%, 1.5%, 2.0%, and 2.5% (v/v).

Sodium hydroxide solutions (% w/v): 0.04%, 0.06%, 0.08%, 0.10%, and 0.15% w/v (about 0.0100, 0.0150, 0.0200, 0.0250, and 0.0375mol L^−1^, respectively).

Sodium bicarbonate solutions (% w/v): 1.0%, 2.0%, 3.0%, 4.0%, and 5.0% w/v (about 0.119, 0.238, 0.357, 0.476, and 0.595mol L^−1^, respectively).

Sodium chloride solutions (% w/v): 0.5%, 1.0%, 1.5%, 2.0%, and 2.5% w/v (about 0.0856, 0.171, 0.257, 0.342, and 0.428mol L^−1^, respectively).

Phosphotungstic acid solutions (mmol L^−1^): 12, 24, 36, 48, and 60mmol L^−1^.

Hydrogen peroxide solutions (mmol L^−1^): 15, 30, 60, 120, and 180mmol L^−1^ (prepared from a 30% H_2_O_2_ stock solution by dilution).

All solutions were freshly prepared prior to the ultrasonic treatment.

### 2.3 Ultrasonic power treatment

Ultrasonic irradiation was performed using a probe-type ultrasonic processor (JY92-IIN, Ningbo Scientz Biotechnology Co., Ltd., Ningbo, China) equipped with a titanium horn (tip diameter: 6 mm). Unless otherwise specified, the experiments were conducted in a cylindrical glass beaker containing 50.0 mL of the test solution. The probe was positioned vertically at the center of the beakers. The distance between the probe tip and the bottom of the beaker was fixed at 1.5 cm, which was used as the standard probe position for all experiments except the “probe position” study.

To minimize the temperature effects, the beaker was immersed in an external water bath, and the bulk temperature was maintained at 20 ± 2°C during sonication (temperature monitored by a thermometer). During sonication, the bulk temperature was monitored at regular intervals (every 2 min) and maintained within 20 ± 2°C using the external water bath. Prior to sonication, the solutions were freshly prepared and equilibrated to the target temperature. After sonication, aliquots were immediately subjected to iodometric analysis (Section 2.4).

**Fig. 1:**
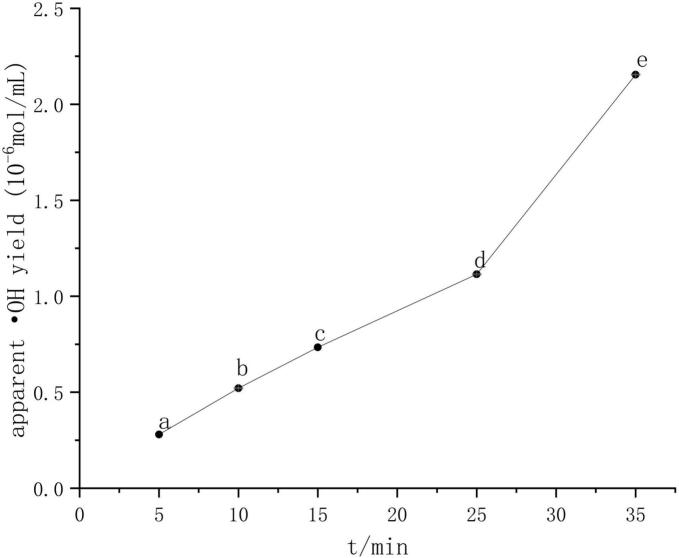
Impact of Power Ultrasonic Treatment Time on Apparent •OH Yield Different letters (a, b, c, d, and e) indicate significant differences between groups (p < 0.05).
(ii) ultrasonic power: 60–300 W (Fig. 2);

In single-factor experiments, one parameter was varied while the others were kept constant at the standard conditions. The investigated ranges were as follows:

(i) sonication time: 5–35 min (Fig. 1);

**Fig. 2:**
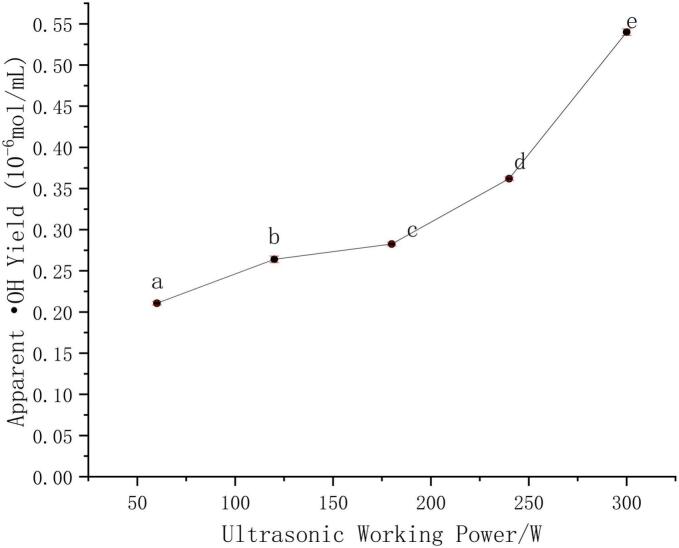
Impact of Ultrasonic Working Power on Apparent •OH Yield Different letters (a, b, c, d, and e) indicate significant differences between groups (p < 0.05).
(iii) duty cycle (pulse mode): 20%–100% (Fig. 3);

**Fig. 3:**
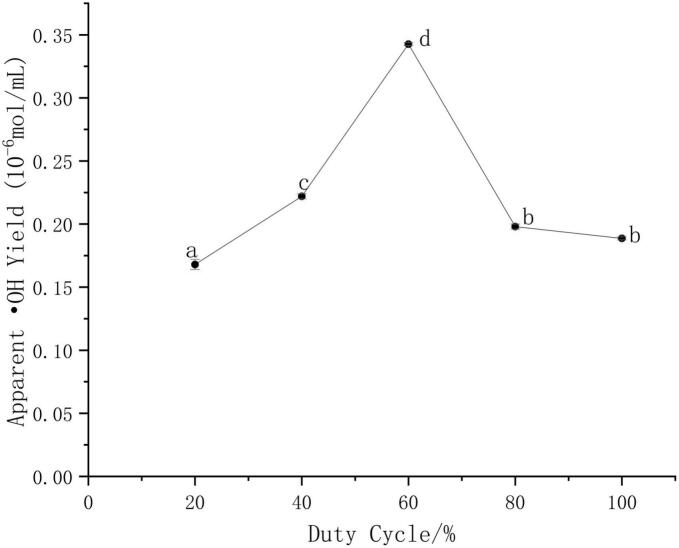
Impact of Duty Cycle on Apparent •OH Yield Different letters (a, b, c, and d) indicate significant differences between groups (p < 0.05).
(iv) probe position:high, middle, and low (Fig. 4). For the probe position study, the probe tip was set at 0.5 cm below the liquid surface (high), at the middle height of the liquid column (middle), or 0.5 cm above the bottom of the beaker (low), while keeping all other conditions unchanged.

**Fig. 4:**
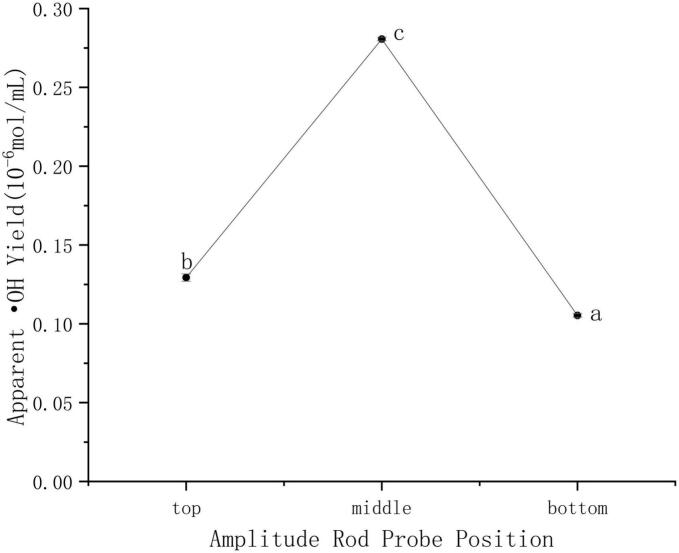
Impact of Amplitude Rod Probe Position on Apparent •OH Yield Different letters (a, b, c) indicate significant differences between the groups (p < 0.05).

For the “different solution systems” experiments, each solution (Section 2.2) was sonicated under standard conditions for 10 or 30 min, as specified in the corresponding figures (Fig. 5, Fig. 6, Fig. 7, Fig. 8, Fig. 9, Fig. 10, Fig. 11, Fig. 12, Fig. 13).

**Fig. 5:**
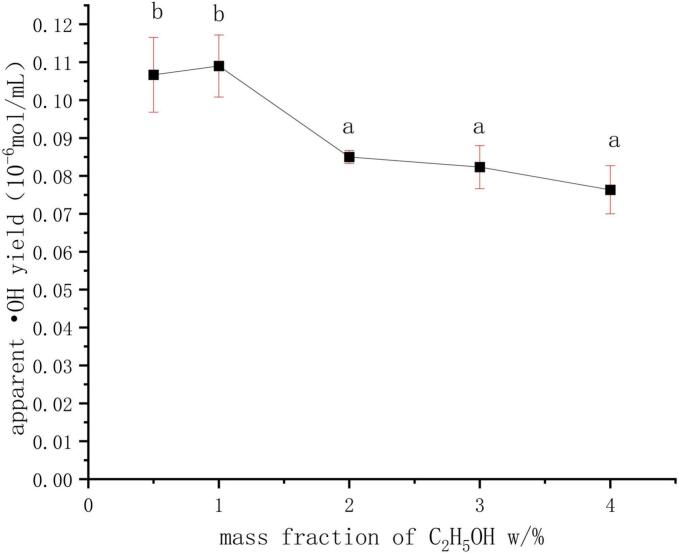
Changes in Apparent •OH Yield in Ethanol Solutions after 10 min of Power Ultrasound Different letters (a and b) indicate significant differences between the groups (p < 0.05).

**Fig. 6:**
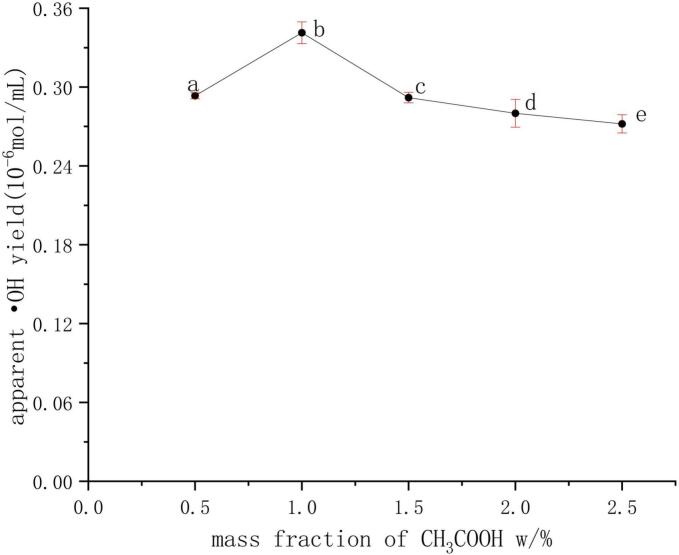
Changes in Apparent •OH Yield in Acetic Acid Solutions after 10 min of Power Ultrasound Different letters a, b, c, d, and e indicate significant differences between groups (p < 0.05).

**Fig. 7:**
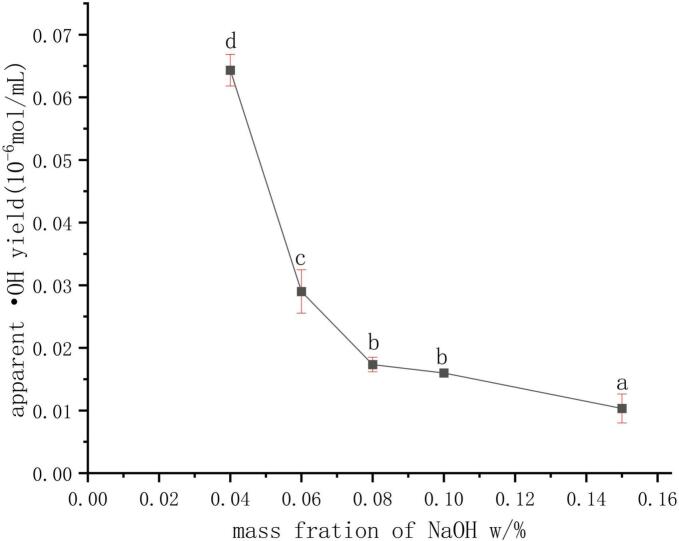
Changes in Apparent •OH Yield in Sodium Hydroxide Solutions after 10 min of Power Ultrasound Different letters (a, b, c, d) indicate significant differences between groups (p < 0.05).

**Fig. 8:**
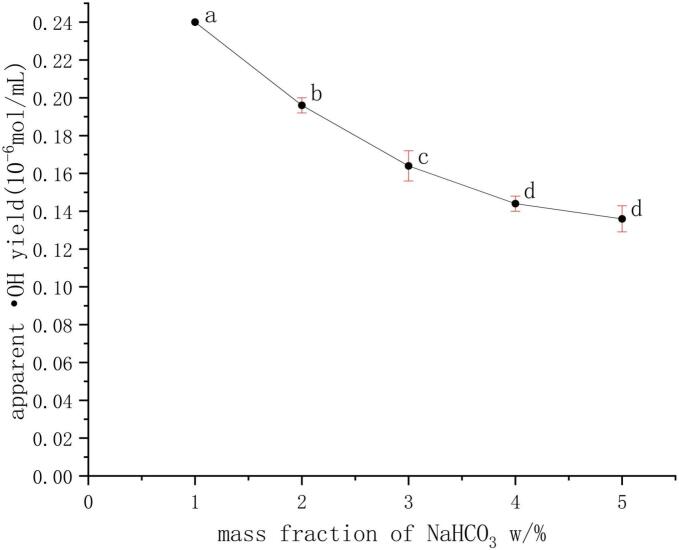
Changes in Apparent •OH Yield in Sodium Bicarbonate Solutions after 10 min of Power Ultrasound Different letters (a, b, c, d) indicate significant differences between groups (p < 0.05).

**Fig. 9:**
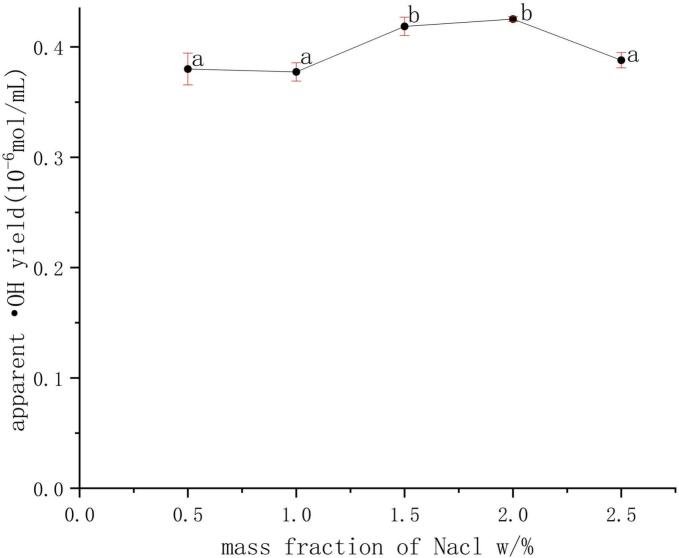
Changes in Apparent •OH Yield in Sodium Chloride Solutions after 10 min of Power Ultrasound Different letters a, b indicate significant differences between groups (p < 0.05).

**Fig. 10:**
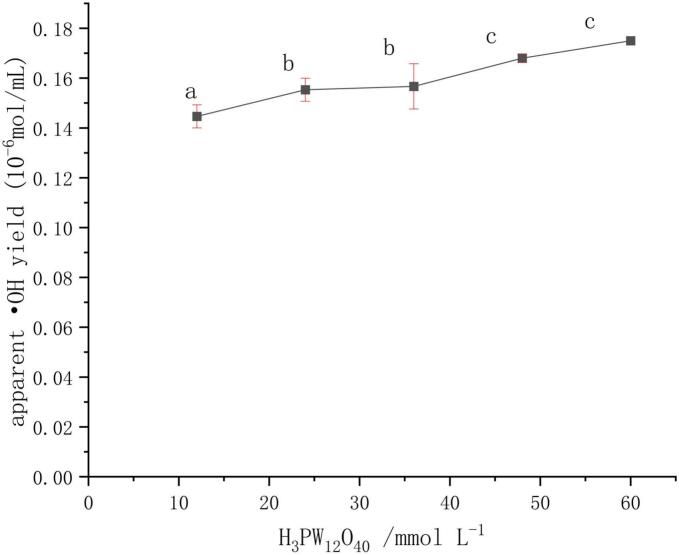
Changes in Apparent •OH Yield in Phosphotungstic Acid Solutions after 10 min of Power Ultrasound Different letters (a, b, c) indicate significant differences between groups (p < 0.05).

**Fig. 11:**
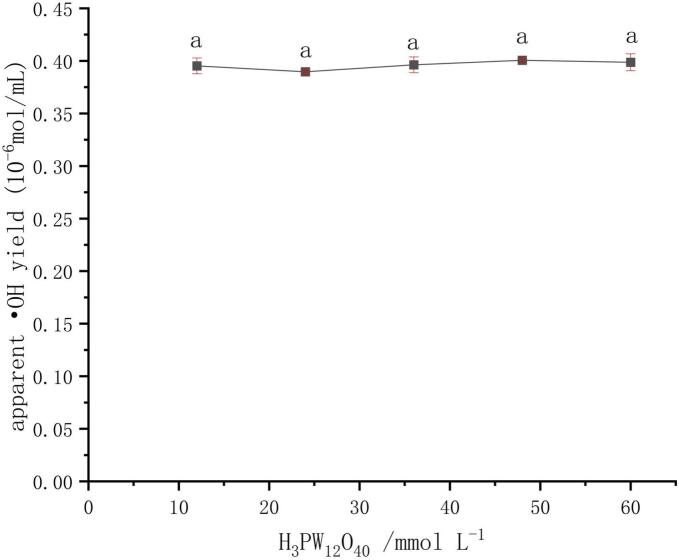
Changes in Apparent •OH Yield in Phosphotungstic Acid Solutions after 30 min of Power Ultrasound Different letters indicate significant differences between the groups (p < 0.05).

**Fig. 12:**
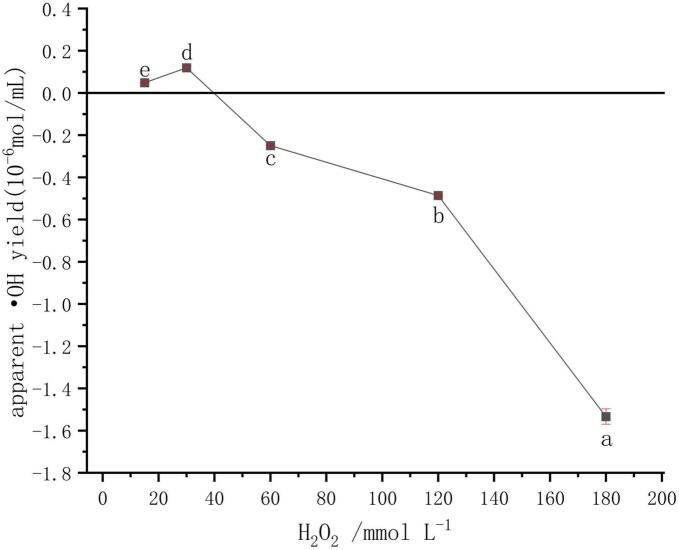
Changes in Apparent •OH Yield in Hydrogen Peroxide Solutions after 10 min of Power Ultrasound Different letters a, b, c, d, and e indicate significant differences between groups (p < 0.05).

**Fig. 13:**
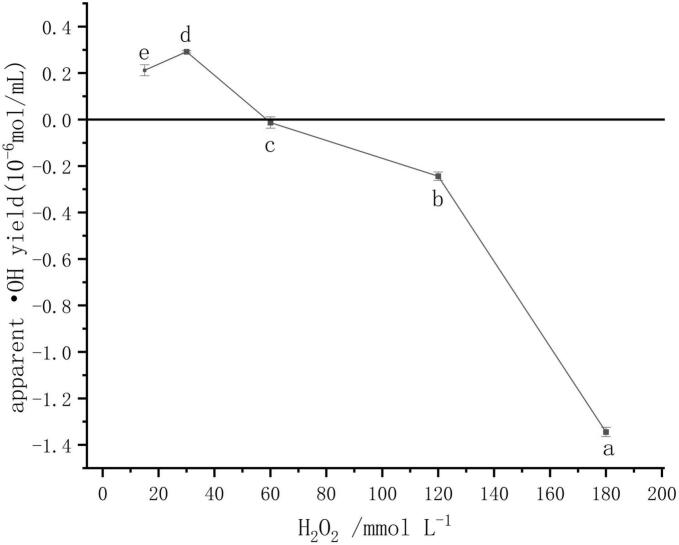
Changes in Apparent •OH Yield in Hydrogen Peroxide Solutions after 30 min of Power Ultrasound Different letters a, b, c, d, and e indicate significant differences between groups (p < 0.05).

### 2.4 Iodometric determination of apparent •OH yield

The hydroxyl radicals generated during ultrasonic irradiation were quantified using an iodometric method based on the oxidation of iodide (I^−^) to iodine (I_2_). In this study, KI was used as the background electrolyte (0.4mol L^−1^) in all test solution systems (Section 2.2). Immediately after sonication, an aliquot of the reaction mixture (Vsample = 10.0 mL) was transferred to an Erlenmeyer flask and kept in the dark for 5 min to complete the reaction. The liberated iodine was titrated with a standardized sodium thiosulfate solution (Na_2_S_2_O_3_, C_t = 0.002mol L^−1^) until the solution turned pale yellow. Subsequently, a starch indicator (1%, w/v) was added, and titration was continued until the colorless endpoint. A blank was prepared and analyzed under the same conditions without ultrasonic irradiation, and the titrant volume was corrected by subtracting the blank consumption. The Na_2_S_2_O_3_ titrant was standardized using KIO_3_ as the primary standard. For each solution system (including each H_2_O_2_ concentration), a matrix-matched blank (same composition without sonication) was analyzed and used for blank correction.

The amount of iodine produced was calculated as follows:

$$n\left(I_{2}\right)=\frac{C_{t}\times \left(V_{t}-V_{b}\right)}{2}$$

where ${\;C}_{t}$(mol L^−1^) is the concentration of Na_2_S_2_O_3_, $V_{t}$(L) is the volume of Na_2_S_2_O_3_ consumed for the sonicated sample, and ${\;V}_{b}$(L) is the volume consumed by the blank. The apparent •OH yield was expressed as follows:

$$\left[\cdot OH\;\right]\left(mol/mL\right)=\frac{n(I_{2})}{Vsample(mL)}$$

and reported as × 10^−6^ mol mL^−1^. All experiments were performed in triplicate, and the results are presented as mean ± standard deviation. It should be emphasized that the iodometric method used here does not measure the instantaneous steady-state concentration of •OH; instead, it reflects the accumulated iodometric response under the specified conditions and is therefore discussed as the apparent •OH yield. In addition, control tests were performed using *tert*-butanol (t-BuOH, 50 mM) as an •OH scavenger in a representative bicarbonate matrix (0.4mol L^−1^ KI + 3% (w/v) NaHCO_3_). t-BuOH (240 µL) was added into 50.0 mL solution, followed by sonication at 180 W and 50% duty cycle for 10 min (probe centered, tip 1.5 cm above the beaker bottom; 20 ± 2°C water bath). A no-ultrasound control was also conducted for a representative PTA system (36mmol L^−1^ PTA in 0.4mol L^−1^ KI), with the probe inserted identically but the generator kept OFF for 10 or 30 min at 20 ± 2°C, to assess non-specific contributions to the iodometric signal.

### 2.5 Statistical analysis

All experiments were performed in triplicates. The data were statistically analyzed for significance using IBM SPSS Statistics software (version 19.0) and graphically represented using Origin 8.0. The main uncertainty contributors in the iodometric measurement include the standardization of the Na_2_S_2_O_3_ titrant, burette reading and endpoint determination, and blank correction (Vt − Vb). To minimize these effects, the titrant was standardized with KIO_3_, matrix-matched blanks were used, and all measurements were performed in triplicate and reported as mean ± standard deviation. A full uncertainty budget analysis is beyond the scope of the present parametric study.

## 3 Results and discussion

### 3.1 Impact of ultrasonic treatment on apparent •OH yield in water

#### 3.1.1 Effect of sonication time on apparent •OH yield

This section investigates the influence of varying sonication durations on the apparent •OH yield in aqueous solutions. The experiments were performed at a fixed ultrasonic power of 180 W and duty cycle of 50%, with the amplitude rod probe centrally positioned in the solution. The selected sonication durations were 5, 10, 15, 25, and 35 min.

As illustrated in Fig. 1, sodium thiosulfate consumption significantly increased with an extended ultrasonic treatment time, indicating a corresponding increase in the apparent •OH yield. Initially, within the 5–25 min interval, a modest increase in sodium thiosulfate consumption was observed; however, a pronounced increase was recorded between 25 and 35 min (p < 0.05). These findings corroborate the observations of Ri-Fu Yanga [16], who reported an enhanced ultrasonic cavitation effect and consequently elevated ·OH production with prolonged sonication. Furthermore, Linzheng Ye [17] noted that the intensification of ultrasonic cavitation effects correlated positively with the increased sonication duration. This trend is consistent with enhanced sonochemical activity under prolonged irradiation. Thus, extending the ultrasonic exposure led to a higher accumulated iodometric response under the present instrument settings.

#### 3.1.2 Effect of ultrasonic power on apparent •OH yield

This section of the study examined the impact of varying ultrasonic power levels on the concentration of free radicals in aqueous solutions, with sonication conducted for 5 min at a 50% duty cycle. The ultrasonic probe was centrally positioned within the solution, and ultrasonic power settings of 60, 120, 180, 240, and 300 W were evaluated.

As illustrated in Fig. 2, increasing the ultrasonic power led to higher sodium thiosulfate consumption, indicating an increased apparent •OH yield. Sodium thiosulfate consumption increased modestly from 60 W to 240 W, with a significant increase observed between 240 W and 300 W (p < 0.05). Shida Chuai [18] previously reported that the cavitation intensity increased with increasing ultrasonic power. Similarly, P.B. Patil [19], who studied the degradation of thiamethoxam via ultrasonic cavitation, found that the cavitation efficiency improved with enhanced ultrasonic power, corroborating the findings of this study. This behavior is often associated with stronger sonochemical effects at higher input settings; however, because no direct acoustic-field measurement was performed, the power dependence reported here is interpreted primarily as an empirical trend in apparent •OH yield under fixed instrument settings. Previous studies have reported enhanced sonochemical activity at elevated ultrasonic power [20], [21], which is consistent with the increased iodometric response observed here.

#### 3.1.3 Effect of ultrasonic duty cycle on apparent •OH yield

This section explores the influence of different duty cycles on apparent •OH yield in aqueous solutions under the conditions of 5 min of sonication at 180 W power. The ultrasonic amplitude probe was centrally positioned, and duty cycles of 20%, 40%, 60%, 80%, and 100% were evaluated.

Fig. 3 shows that the sodium thiosulfate consumption, and thus the apparent •OH yield, initially increased with the duty cycle, reached a peak, and subsequently decreased. Specifically, sodium thiosulfate consumption increased from 2.77 mL at a 40% duty cycle to 4.27 mL at 60%, and then decreased to 2.47 mL at an 80% duty cycle. These changes were statistically significant, showing an increase of 40%-60% (p < 0.05) and a decrease of 60%-80% (p < 0.05). According to HengLein [22], cavitation effectiveness depends on the time required for cavitation bubbles to reach an optimal size and the rate of their migration toward the pressure antinodes in the acoustic standing wave field. At lower duty cycles, the individual pulses were sufficiently spaced to minimize the mutual interference. Increasing the duty cycle extends the active ultrasound period, which initially enhances cavitation until saturation occurs, thereby diminishing the effectiveness of subsequent pulses. An excessive duty cycle reduces the interval between pulses, causing bubbles to collapse prematurely and preventing optimal cavitation. Therefore, the apparent •OH yield initially increased with duty cycle, peaked at intermediate duty cycles, and subsequently decreased at higher duty cycles.

#### 3.1.4 Effect of ultrasonic probe position on apparent •OH yield

This study examined the impact of ultrasonic probe positioning on apparent •OH yield in aqueous solutions under defined ultrasonic conditions (5 min sonication time, 180 W power, and 50% duty cycle). The probe was positioned at three distinct locations: top (0.5 cm from the liquid surface), middle (1.5 cm above the beaker bottom), and bottom (0.5 cm above the beaker bottom).

As shown in Fig. 4, the highest consumption of sodium thiosulfate (3.53 mL) occurred when the probe was positioned in the middle of the solution, whereas the lowest consumption (1.32 mL) occurred when the probe was positioned at the bottom. The top position yielded an intermediate consumption value of 1.60 mL. Therefore, transitioning the probe from the top to the bottom of the solution initially led to an increase in the iodometric response, followed by a decrease. Statistical analysis revealed a significant increase in sodium thiosulfate consumption when moving from the top to middle position (p < 0.05) and a significant decrease when moving from the middle to bottom position (p < 0.05). Peilin Cao [23] previously investigated the effects of probe positioning on cavitation intensity and observed the maximum cavitation energy at the midpoint, with diminishing intensity at positions closer to or farther from this optimal point. In the present setup, positions close to the liquid surface or the rigid bottom may lead to less uniform acoustic conditions and altered bubble dynamics, which could affect the accumulated iodometric response. Importantly, probe position can also influence acoustic impedance and standing-wave patterns, potentially changing the effective acoustic power delivered to the liquid; therefore, without calorimetric power measurements, the position dependence reported here should be interpreted as an empirical trend under fixed instrument settings rather than a direct measure of cavitation intensity.

### 3.2 Influence of power ultrasound on apparent •OH yield in different solution systems

#### 3.2.1 Impact of ethanol concentration on apparent •OH yield

This section investigates the influence of varying ethanol concentrations on apparent •OH yield under controlled ultrasonication conditions. Ethanol solutions with 0.5%, 1%, 2%, 3%, and 4% (v/v) were sonicated for 10 min at 180 W power, 50% duty cycle, and with the probe positioned at the center.

Fig. 5 shows that increasing the ethanol concentration from 0.5% to 4.0% generally resulted in a gradual decline in apparent •OH yield. No statistically significant differences in the apparent •OH yield were detected between ethanol concentrations of 0.5%-1.0% and 2.0%-4.0% (p > 0.05), implying negligible changes in the radical concentrations within these ranges. However, a significant reduction in apparent •OH yield occurred when the ethanol concentration increased from 1.0% to 2.0% (p < 0.05), demonstrating a distinct inhibitory effect on apparent •OH yield at these concentrations. These findings align with those of Zhang et al. [24], who reported the radical-quenching capability of ethanol solutions and noted enhanced quenching effects at elevated ethanol concentrations. This trend is consistent with rapid reaction and competition of ethanol with reactive species, which reduces the iodometric response and thus the apparent •OH yield as ethanol concentration increases.

#### 3.2.2 Influence of acetic acid concentration on apparent •OH yield

To evaluate the impact of ultrasonic treatment on apparent •OH yield in acetic acid solutions, preliminary experiments were conducted to establish a suitable concentration range. The acetic acid concentrations investigated were 0.5%, 1.0%, 1.5%, 2.0%, and 2.5%. Each solution was subjected to sonication for 10 min at an ultrasonic power of 180 W and a duty cycle of 50%, with the ultrasonic probe centrally positioned within the solution.

As shown in Fig. 6, the apparent •OH yield initially increased and subsequently decreased with an increase in acetic acid concentration. Specifically, increasing the acetic acid concentration from 0.5% to 1.0% significantly enhanced iodometric response (p < 0.05). Conversely, a further increase in acetic acid concentration from 1.0% to 1.5% significantly reduced the formation of radicals (p < 0.05). No significant variation in apparent •OH yield was observed between 1.5% and 2.5% concentrations. These findings suggest that an optimal acetic acid concentration between 0.5% and 1.5% exists for maximizing ·OH production. Consistent with the findings reported by Rui Yangxiao [25], the initial enhancement at moderate acetic acid levels may be associated with changes in cavitation conditions that influence the accumulated oxidative response. At higher acetic acid concentrations, the response decreased and then approached a plateau, which may reflect combined effects of altered cavitation behavior and matrix-dependent pathways in the KI system. Importantly, acetic acid can react rapidly with •OH and thus compete with iodide for reactive species, reducing the iodometric response at sufficiently high concentrations rather than literally “removing radicals” from the system [26].

#### 3.2.3 Influence of sodium hydroxide concentration on apparent •OH yield

This experiment evaluated the impact of sodium hydroxide concentration on the iodometrically determined •OH yield under controlled ultrasonication conditions. Sodium hydroxide solutions with concentrations of 0.04%, 0.06%, 0.08%, 0.10%, and 0.15% were prepared and sonicated for 10 min at 180 W power, 50% duty cycle, and with the probe positioned at the center of the solution.

The results presented in Fig. 7 indicate a general downward trend in apparent •OH yield with increasing sodium hydroxide concentrations ranging from 0.04% to-0.15%. A statistically significant decrease in radical concentration was observed within the concentration ranges of 0.04%-0.08% and 0.10%-0.15% (p < 0.05). However, no significant difference was noted between 0.08% and 0.10% (p > 0.05), suggesting the stabilization of •OH production within this range. Notably, NaOH is not expected to directly “quench” •OH through reaction with Na^+^, and reactions involving OH^-^mainly reflect changes in radical pathways rather than simple radical removal. Therefore, the observed decrease is more reasonably attributed to pH-induced effects that can influence cavitation behavior and radical speciation, as well as the iodometric response (iodine speciation and competing reactions in the KI system). In addition, at the concentrations tested, changes in ionic strength may also contribute to the observed trend. Overall, increasing alkalinity led to a lower apparent iodometric signal, which is discussed here as a reduced accumulated oxidative equivalent under the specified conditions. A similar inhibitory trend under alkaline conditions has been reported in previous sonochemical studies [27], although the underlying mechanism is likely dominated by pH-and matrix-dependent effects rather than direct scavenging of •OH.

#### 3.2.4 Influence of sodium bicarbonate concentration on apparent •OH yield

This study investigated the influence of varying sodium bicarbonate concentrations on apparent •OH yield under specific ultrasonication conditions. Sodium bicarbonate solutions with w/v concentrations of 1%, 2%, 3%, 4%, and 5% were prepared and sonicated for 10 min at an ultrasonic power of 180 W and a duty cycle of 50%, with the probe positioned at the center of the solution.

As illustrated in Fig. 8, the apparent •OH yield generally exhibited a declining trend with increasing sodium bicarbonate solution concentrations. Specifically, a significant decrease (p < 0.05) was observed when the sodium bicarbonate concentration was increased from 1% to 3%, indicating the suppressive effect of bicarbonate ions on radical formation. However, increasing the concentration from 4% to 5% did not result in any significant changes. Importantly, bicarbonate/carbonate species do not simply “remove” radicals; instead, they convert •OH into secondary radicals. In aqueous systems, •OH can react with HCO3^−^/CO32^−^ to form the carbonate radical anion (CO3•^−^). This secondary radical remains an oxidizing species and may also contribute to iodide oxidation, which complicates the direct interpretation of the iodometric signal as solely reflecting •OH. In addition, sodium bicarbonate increases alkalinity and ionic strength, which can influence cavitation behavior and radical pathways. Therefore, the observed decrease in the iodometric response is more appropriately discussed as a reduced apparent •OH yield under bicarbonate-containing conditions, rather than a literal reduction in “total free radicals” in the system. A related radical-driven reaction environment has been discussed in previous work [28], however, the present observations are interpreted in the context of aqueous sonochemical reactions and iodometric response. In the t-BuOH control (0.4mol L^−1^ KI + 3% NaHCO_3_), the blank-corrected iodometric signal decreased to approximately 10% of the value obtained without t-BuOH, indicating that the response under these conditions is predominantly •OH-driven, with a minor residual non-specific contribution.

#### 3.2.5 Influence of sodium chloride concentration on apparent •OH yield

This section investigates the effect of variations in sodium chloride concentration on apparent •OH yield under controlled ultrasonic conditions. Sodium chloride solutions with w/v concentrations of 0.5%, 1.0%, 1.5%, 2.0%, and 2.5% were sonicated for 10 min at an ultrasonic power of 180 W and a duty cycle of 50%, with the probe positioned at the center of the solution.

As depicted in Fig. 9, no significant variation in apparent •OH yield was noted between 0.5% and 1.0% sodium chloride concentrations, suggesting a minimal impact at lower concentrations. A significant increase (p < 0.05) in apparent •OH yield was observed when the sodium chloride concentration was increased from 1.0% to 1.5%, indicating an enhanced iodometric response. Subsequently, no statistically significant changes were detected between 1.5% and 2.0%; however, a notable decrease (p < 0.05) was observed at the highest tested concentration of 2.5%. The absence of a significant difference between 1.5% and 2.0% concentrations may indicate an optimal range for the iodometric response within this interval. Isil Gultekin [29] previously reported that chloride ion concentrations below 1250 mM resulted in a gradual decrease in apparent •OH yield, negatively impacting the efficiency of dye photodecolorization processes, which is consistent with present findings. The initial enhancement may be related to changes in ionic strength and cavitation behavior, whereas the reduction at higher chloride concentrations is likely associated with competing reactions and matrix effects in the KI system, thereby lowering the apparent •OH yield.

#### 3.2.6 Influence of phosphotungstic acid concentration on apparent •OH yield

This section evaluates the variation in apparent •OH yield with different phosphotungstic acid concentrations under specific ultrasonic conditions. Solutions of phosphotungstic acid were prepared at concentrations of 12, 24, 36, 48, and 60 mmol/L and sonicated for 10 and 30 min at 180 W power and 50% duty cycle, with the probe positioned at the center of the solution.

As shown in Fig. 10, during 10 min of sonication, the apparent •OH yield exhibited a general upward trend corresponding to increasing phosphotungstic acid concentrations from 12 to 60 mmol/L. Statistically significant increases in the apparent •OH yield were observed between 12 and 24 mmol/L and 36 and 48 mmol/L (p < 0.05). However, no significant variations were detected within the ranges of 24–36 mmol/L and 48–60 mmol/L (p > 0.05). This trend is consistent with reports that PTA-containing systems can enhance the overall oxidative response under ultrasound by Liyan Liu [30]. However, PTA may also contribute to iodide/iodine chemistry via redox cycling; therefore, in the present work the PTA effect is interpreted primarily in terms of increased iodometric yield rather than being attributed exclusively to •OH.

Fig. 11 illustrates the results obtained after a 30-minute sonication period, revealing no significant difference in apparent •OH yield across phosphotungstic acid concentrations ranging from 12 mmol/L to 60 mmol/L (p > 0.05). Dharmendra Kumar Bal [31] similarly demonstrated that the difference in apparent •OH yield diminished gradually over prolonged ultrasonic exposure, consistent with our experimental observations. This behavior may reflect a tendency toward a plateau in the accumulated iodometric response under the present conditions. To further assess non-specific contributions, a no-ultrasound control was conducted for PTA (36 mmol/L in 0.4 mol/L KI) with the probe inserted identically but the generator kept OFF. A measurable iodometric signal was observed even without ultrasound, while ultrasound produced a clearly stronger signal; therefore, PTA-related effects are interpreted primarily in terms of iodometric yield and overall oxidative response rather than being attributed exclusively to •OH.

#### 3.2.7 Influence of hydrogen peroxide concentration on apparent •OH yield

This section examines the pattern of apparent •OH yield variations at different H_2_O_2_ concentrations under defined ultrasonic parameters·H_2_O_2_ solutions at concentrations of 15, 30, 60, 120, and 180 mmol/L were sonicated for 10 and 30 min under ultrasonic conditions of 180 W power and 50% duty cycle, with the ultrasonic probe centrally positioned.

Fig. 12, Fig. 13 demonstrate that for both 10 and 30 min sonication durations, the apparent •OH yield initially increased with increasing H_2_O_2_ concentration, peaking at 30 mmol/L, before subsequently declining with further increases in concentration up to 180 mmol/L. Notably, significant increments in apparent •OH yield were identified between 15 mmol/L and 30 mmol/L (p < 0.05), whereas significant reductions were observed between 30 mmol/L and 180 mmol/L (p < 0.05). This observation is consistent with reports on the concentration-dependent role of H_2_O_2_ in sonochemical reactions[32], which noted an initial enhancement in ·OH radical generation with increased H_2_O_2_ concentration, followed by a decline in target compound degradation at excessively high concentrations. P.B. Patil [19] reported analogous results, demonstrating a similar initial increase, followed by a decrease in thiamethoxam removal efficiency with increasing H_2_O_2_ levels. This phenomenon occurs because H_2_O_2_ can enhance the oxidative response under ultrasound at moderate concentrations, whereas at higher concentrations self-decomposition, competing reactions, and iodine speciation effects can reduce the apparent •OH yield. Notably, negative values were observed at high H_2_O_2_ concentrations after both 10 min and 30 min sonication. This does not indicate a physically negative apparent •OH yield; rather, it arises from the blank-corrected iodometric signal (Vt-Vb) becoming negative, that is, the titratable iodine in the sonicated samples was lower than that in the corresponding non-sonicated controls. This behavior is attributed to matrix-induced interference and competing side reactions in the KI/H_2_O_2_ system under ultrasonication (including accelerated H_2_O_2_ decomposition and altered iodine speciation), which can reduce the measurable iodometric response. Therefore, negative values were regarded as below the effective detection limit and interpreted as approximately zero in the discussion.

Overall considerations on iodometric response, radical kinetics and matrix effects. Hydroxyl radicals (•OH) are extremely short-lived and typically exist at very low steady-state concentrations in aqueous sonochemical systems. Therefore, the present iodometric approach should not be interpreted as a direct measurement of instantaneous [•OH]. Rather, the titrated I_3_^-^ signal represents an accumulated oxidation response (titrable iodine) integrated over the sonication period, which is influenced not only by •OH formation but also by competing reactions and iodine speciation. In addition to •OH, other oxidizing species and radical-derived intermediates (e.g., HO_2_•/O_2_•^-^ or secondary radicals generated from additives) may contribute to iodide oxidation, while some matrix components may decrease the measurable iodine through side reactions. Consequently, the results are discussed as an apparent/accumulated •OH yield under defined conditions. From a kinetic perspective, the impact of added solutes can be rationalized by competition for •OH. Many additives (e.g., ethanol, acetate/bicarbonate-related species) react with •OH at diffusion-controlled rates; hence increasing their concentrations is expected to divert •OH away from iodide and alter the apparent iodometric response. Importantly, “scavenging” does not necessarily remove radicals from the system; it often converts one radical into another. For example, reactions of •OH with bicarbonate/carbonate generate carbonate radical anions (CO_3_•^-^), which remain oxidizing and may also oxidize iodide, potentially complicating interpretation. Likewise, changes in pH introduced by NaOH, acetic acid, bicarbonate, or phosphotungstic acid can affect cavitation behavior and radical pathways, and can also influence iodine equilibria (I_2_/I_3_^-^) and analytical response. Furthermore, at the relatively high solute levels used here, changes in physicochemical properties (ionic strength, viscosity, surface tension, and vapor pressure) may modify cavitation intensity and mass transfer, beyond purely kinetic scavenging effects. In H_2_O_2_^-^containing systems, the dual role of H_2_O_2_ should be considered: moderate H_2_O_2_ can promote oxidative capacity, while at higher concentrations it can participate in well-known radical chain/competition reactions and alter iodine speciation, leading to a reduced or even negative blank-corrected iodometric signal. These considerations define the scope and limitations of the present iodometric evaluation and provide a consistent framework for interpreting matrix-dependent trends. A further limitation of this study is that we did not perform calorimetric power measurements or direct acoustic field characterization; thus, input power and probe position may influence not only cavitation activity but also actual energy coupling to the liquid. Nevertheless, all experiments were conducted on the same setup under controlled geometry and temperature, enabling consistent comparison of relative iodometric trends across conditions.

## 4 Conclusions

In this study, an ultrasonic device equipped with an amplitude rod of 6 mm diameter was employed, and the iodometric method was utilized to examine variations in the apparent •OH yield under different ultrasonic treatment conditions and in various solution systems. The apparent •OH yield in the aqueous solutions increased with extended sonication time and elevated ultrasonic power. The iodometric response initially increased and subsequently decreased as the ultrasonic duty cycle increased, peaking at a 60% duty cycle. Additionally, the positioning of the amplitude rod probe significantly influenced the apparent •OH yield; as the probe was moved downwards from the top to the bottom of the solution, the response increased to a maximum at the midpoint and then declined. Under the tested conditions, ethanol, sodium hydroxide, and sodium bicarbonate generally decreased the apparent •OH yield, whereas acetic acid exhibited a biphasic trend with an initial increase followed by a decrease at higher concentrations. Sodium chloride showed a concentration-dependent effect, with the highest apparent response observed at 1.5%–2.0% (w/v) under the present conditions. In phosphotungstic acid solution systems, the iodometric response increased with PTA concentration at 10 min, whereas the concentration-dependent differences diminished after prolonged sonication (30 min); therefore, PTA-related effects are interpreted in terms of overall iodometric yield response rather than being attributed exclusively to •OH. Finally, hydrogen peroxide exhibited a nonlinear effect, enhancing the apparent •OH yield at moderate concentrations but reducing it at higher concentrations due to competing reactions and matrix effects in the KI/H_2_O_2_ system.

Collectively, this study demonstrates that ultrasonic operating parameters and solution composition distinctly affect the accumulated iodometric response in probe-ultrasonic systems. The results provide practical guidance for regulating oxidative response through adjustment of sonication conditions and solution additives, while acknowledging the scope and limitations of KI iodometry in additive-containing matrices.

## Fundings

This study was supported by the Scientific Research Foundation for Doctors of Zhengzhou Normal University (Grant number: 702457), Henan Province General Education Model Courses (Grant number: PX-99232277), the 2026 Zhengzhou Normal University's Innovation Training Program (Grant number: DCY2026003), the 2025 Henan Provincial College Students’Innovation Training Program (Grant number S202512949015), the Science and Technology Research Program of Henan Province (Grant number: 2178), the Key Scientific Research Projects of Colleges and Universities in Henan Province (Grant number: 25B530004).

## CRediT authorship contribution statement

**Yuanfang Liu:** Conceptualization. **Yuanxiao Liu:** Formal analysis. **Hailu Hou:** Data curation. **Minghui Liu:** Investigation. **Ying-Ying Li:** Methodology. **Jinming Xu:** Software. **Jiayu Guo:** Validation.

## Declaration of competing interest

The authors declare that they have no known competing financial interests or personal relationships that could have appeared to influence the work reported in this paper.

## Data Availability

Data will be made available on request.
